# Supplementary material for: Natural Compounds for Bone Remodeling: A Computational and Experimental Approach Targeting Bone Metabolism-Related Proteins
Source: Int J Mol Sci. 2024 May 6;25(9):5047. doi: 10.3390/ijms25095047 (PMC11084538; doi:10.3390/ijms25095047)
Supplement: Supplementary file 1 [file ijms-25-05047-s001.zip › ijms-2942473-supplementary.pdf]

International Journal of
Molecular Sciences

MDPI

Article

Natural Compounds for Bone Remodeling: A Computational and Experimental Approach Targeting Bone Metabolism-Related Proteins

Alexandros-Timotheos Loukas [1,2,†], Michail Papadourakis [2,*,†], Vasilis Panagiotopoulos [2,3], Apostolia Zarmpala [2], Eleni Chontzopoulou [2], Stephanos Christodoulou [2], Theodora Katsila [4], Panagiotis Zoumpoulakis [1,4] and Minos-Timotheos Matsoukas [2,3,*]

1 Department of Food Science and Technology, University of West Attica, Ag. Spyridonos, 12243 Egaleo, Greece
2 Cloudpharm Private Company, Kifissias Avenue 44, 15125 Marousi, Greece
3 Department of Biomedical Engineering, University of West Attica, Ag. Spyridonos, 12243 Egaleo, Greece
4 Institute of Chemical Biology, National Hellenic Research Foundation, 11635 Athens, Greece
* Correspondence: mpapadourakis@cloudpharm.eu (M.P.); mmatsoukas@uniwa.gr (M.-T.M.); Tel.: +30-538-5364 (M.P. & M.-T.M.)
† These authors contributed equally to this work.

2. Results

2.1. Selection of the most promising natural compounds for MD simulations

Table S1. Docking scores of the 187 natural compounds on p38b and ERK1

Names	Docking scores p38b (kcal/mol)	Docking scores ERK1 (kcal/mol)
Pratensein	-7.7	-8.8
3’O-methylorobol	-9.1	-8.6
6,8-diprenylgenistein	-8.4	-10.2
Afzelin	-8.1	-9.9
Kaempferitrin	-9.3	-9.1
viscumneoside IX	-9.4	-9.3
(-)-epicatechin	-9.1	-8.4
(2S)-2'-methoxykurarinone	-7.8	-8.9
(7R,8S)-ceplignan	-8.4	-8.1
(7R,8S)-dehydrodiconiferyl alcohol	-7.3	-8.0
(7R,8S)-ficusal	-6.8	-6.7
(S)-naringenin 7-O-beta-D-glucoside / Prunin	-8.7	-9.2
1,2-dihydroxy-3-methylanthraquinone	-8.3	-8.9

1,3,8-trihydroxy-2-methoxyanthraquinone	-7.8	-8.5
15,16-dihydrotanshinone I	-9.5	-10.0
2,3,5,4'-Tetrahydroxystilbene-2-O- β -D-glucoside (TSG)	-7.1	-7.9
20(S)-ginsenoside Rh2	-8.9	-8.2
2-hydroxy-1-methoxy-anthraquinone	-7.9	-8.5
2-methoxyanthraquinone	-8.3	-8.4
3-methoxynobiletin	-7.2	-7.5
3-O-beta-D-glucopyranosyl-7-O-alpha-L-arabinofuranosyl-kaempferol	-8.8	-8.4
5-hydroxymethyl-2-furaldehyde	-4.8	-4.5
6-prenylgenistein	-9.0	-9.2
7-O-methyl-luteone	-8.3	-8.7
8-hydroxy-2,6-dimethyl-2-octenoic acid	-6.3	-6.1
8-prenylgenistein	-9.6	-9.8
8-prenylkaempferol	-8.9	-9.0
Acerogenin A	-9.3	-9.0
Acteoside	-8.8	-10.1
Albiflorin	-8.7	-9.1
Apigenin	-9.2	-8.5
Apocynin	-6.1	-5.9
Apomorphine Hydrochloride	-8.4	-9.5
Asperosaponin VI	-9.2	-8.6
Astragalin	-8.1	-8.6
Aucubin	-7.7	-7.3
aureusidin	-9.4	-8.6
avenacoside B	-6.1	-7.4
Bacoside A	-8.2	-8.0
Bakuchiol	-8.3	-8.4

Baohuoside-I	-8.7	-9.6
Bavachalcone	-9.2	-9.3
bavachin	-9.9	-9.7
b-carotene	-11.9	-9.1
Berberine	-9.5	-8.9
Bergapten	-7.3	-7.2
Betulin-3beta-yl-caffeate	-8.9	-10.5
Betulinic acid	-8.3	-9.0
Cajanin	-8.9	-8.5
Catalpol	-8.0	-7.8
chlorogenic acid	-8.4	-8.4
Cholecalciferol	-10.8	-9.1
cimicidanol-3-O-beta-xyloside	-7.7	-8.3
Cimicidol-3-O-beta-xyloside	-7.9	-9.4
Cordycepin	-6.9	-7.0
Corylin	-10.8	-9.2
Costunolide	-7.9	-8.1
cryptotanshinone	-9.6	-10.0
cyanidin-3-O-glucoside	-8.1	-9.0
cyanidin-3-O-rutinoside	-9.5	-9.9
Cyanocobalamin	-8.0	-7.1
Daidzein	-9.0	-8.6
delphinidin-3-O-glucoside	-8.8	-9.4
Delphinidin-3-rutinoside	-10.2	-9.9
delta-tocotrienol	-10.2	-10.5
Deoxyactein	-10.3	-8.4
Dioscin	-7.3	-8.9
Diosgenin	-9.5	-10.1
Diosmetin	-9.3	-8.7
Diospongin B	-9.3	-9.2
Docosahexaenoic Acids	-8.0	-7.3
Echinacoside	-8.7	-9.2
Echinocystic acid	-9.2	-7.7
EGCG	-8.9	-9.5
eicosapentaenoic acid	-7.5	-7.7
Emodin	-8.1	-8.9
Epimedin B	-7.6	-8.8
Epimedin C	-7.9	-8.6
Equol	-9.4	-8.3
Ergocalciferol	-10.3	-9.3
Estrogen	-11.5	-8.8

Estrogens, Conjugated	-10.0	-9.2
Ferulic acid	-6.7	-6.3
Ferutinin	-8.0	-9.0
Folic Acid	-9.7	-9.5
Formononetin	-8.9	-8.5
Gastrodin	-7.4	-6.9
Genipin	-6.8	-6.3
Geniposide	-7.1	-7.7
Geniposidic acid	-7.1	-7.4
Genistein	-9.0	-8.7
Ginsenoside Rb1	-7.7	-7.7
Ginsenoside Rd	-8.1	-8.7
Ginsenoside Rg1	-8.3	-9.0
Ginsenoside Rg3	-6.9	-8.5
Ginsenoside Rb2	-8.4	-6.9
Glycyrrhizic acid	-8.2	-9.0
Hesperidin	-9.2	-9.9
Honokiol	-8.8	-8.1
Hydroxytyrosol	-6.1	-6.1
Hymenialdisine	-8.4	-9.0
Icariin	-7.9	-9.5
Icaritin	-8.5	-8.7
Ikarisoside A	-8.6	-9.9
Imperatorin	-7.8	-8.3
Isoformononetin	-8.9	-8.6
Isoliquiritigenin	-8.9	-8.2
isopsoralen,Angelicin	-7.9	-7.5
isoswertisin-2''-O-rhamnoside	-8	-9.4
isovitexin-2''-O-arabinoside	-8.7	-8.9
kaempferol	-9.4	-8.5
Kirenol	-8.2	-7.4
Kobophenol A	-5.0	-7.7
Kurarinone	-8.6	-9.6
kushenol F	-8.8	-9.5
Laburnetin	-8.8	-9.0
Ligustroside	-8.2	-8.2
Linolenic acid	-6.7	-7.1
loganin	-7.6	-7.6
Luteolin	-9.2	-8.8
luteolin 7-O-beta-D-neohesperidoside / Lonicerin	-9.2	-9.1

Luteone	-9.4	-8.7
Lyciumin A	-8.0	-9.6
Lyciumin B	-7.1	-8.9
Lyciumoside III	-9.0	-8.7
maltol glucoside	-7.5	-6.7
Maohuoside A	-8.0	-9.3
matairesinol	-8.9	-7.9
Melatonin	-7.6	-7.2
Naringenin	-8.9	-8.4
Naringin	-10.0	-9.1
Neobavaisoflavone	-9.9	-10.0
Neoeriocitrin	-10.1	-9.4
nu(e)zhenide	-8.1	-8.5
Oleanolic acid	-9.0	-8.4
Oleanolic acid acetate	-10.1	-8.1
oleoside dimethyl ester	-7.1	-7.1
Oleuropein	-8.6	-8.6
Ophiopogonin D	-7.5	-9.1
Osthole	-7.8	-7.7
palmitic acid	-6.0	-6.3
phloretin	-8.2	-8.2
Phloridzin	-8.5	-8.7
physicion	-8.3	-9.1
pinoresinol	-9.3	-8.4
Pinoresinol diglucoside	-8.0	-8.3
Piperitol	-10.4	-9.3
Podocarnone	-9.9	-9.3
Poncirin	-8.9	-8.6
Psoralen	-7.8	-7.4
Psoralidin	-9.7	-9.8
Puerarin	-8.3	-8.5
Quercetin	-8.3	-8.5
quercetin-7-O-D-glucopyranoside	-9.3	-9.0
Resveratrol	-8.6	-8.1
Rubiadin	-8.2	-8.8
Rubiadin-1-methyl ether	-8.2	-8.7
Rutin	-8.7	-9.9
Sagittatoside A	-8.5	-8.0
Salidroside	-7.7	-7.3
Salvianic acid A	-10.2	-9.6

Salvianic acid B	-8.1	-8.1
Salvianolic acid B	-9.3	-7.7
Samwinol	-8.3	-9.0
Schisandrin A	-6.1	-7.0
scopoletin	-7.0	-6.8
sesaminone	-9.2	-9.2
sophoraflavanone G	-8.1	-8.7
Sophoricoside	-9.1	-10.2
Stachydrine	-4.8	-4.6
Sulforaphane	-4.2	-4.2
Sweroside	-7.4	-8.0
syringaresinol	-8.5	-7.9
Syringic acid	-5.2	-6.1
tanshinone I	-9.3	-10.1
Tanshinone IIA	-10.0	-10.2
tanshinone VI	-8.9	-9.3
Taxifolin	-9.0	-8.5
Tilianin	-9.9	-9.7
Tyrosol	-5.6	-5.4
Ugonin K	-9.5	-9.8
Ursolic acid	-8.9	-8.2
Vanillic acid	-5.9	-5.8
viscumneoside I	-10.0	-8.7
Wedelolactone	-8.4	-9.0
Wighteone	-9.7	-9.3
Xanthogalenol	-8.8	-9.1

Table S2. Natural compounds that have been assessed by the EFSA organization.

Compound	EFSA Scientific Opinion	EFSA panel conclusions	EFSA scientific opinion reference
Naringin	Scientific Opinion on the safety and efficacy of naringin when used as a sensory additive for all animal species	The FEEDAP Panel confirms that the use of naringin under the current authorised conditions of use is safe for the target species, the consumers, and the environment.<br><br>Naringin does not cause severe irritation or corrosion to eyes, is	[1], [2]

		not irritant to the skin and is not classified as a dermal sensitiser.	
Cyanocobalamin	Safety of vitamin B12 (in the form of cyanocobalamin) produced by Ensifer adhaerens CNCM-I 5541 for all animal species, Scientific Opinion on Dietary Reference Values for cobalamin (vitamin B12)	The FEEDAP Panel concluded that cyanocobalamin produced by fermentation with E. adhaerens CNCM-I 5541 is considered safe for all animal species. The use of cyanocobalamin in animal nutrition is of no concern for consumer safety. Cyanocobalamin is non-irritant to skin and non-irritant to eyes. No conclusions could be drawn on the potential of the additive to be a skin sensitiser. The potential endotoxin activity of the additive is unlikely to represent a hazard for users. The use of the additive under assessment in animal nutrition is considered safe for the environment. Cyanocobalamin produced by E. adhaerens CNCM-I 5541 is effective in meeting animal's nutritional requirements when administered via feed	[3]
Rutin	Scientific Opinion on the substantiation of health claims related to rutin and improvement of endothelium-dependent vasodilation (ID 1649, 1783) and protection of DNA, proteins and lipids from oxidative damage (ID 1784) pursuant to Article 13(1) of Regulation (EC) No 1924/2006	The food constituent that is the subject of the health claims is rutin. The Panel considers that rutin is sufficiently characterised	[4]
Resveratrol	Safety of synthetic trans-resveratrol as a novel food pursuant to Regulation (EC) No 258/97	The Panel concludes that the novel food, synthetic trans-resveratrol, is safe under the proposed conditions of use	[5]
Folic Acid	Scientific Opinion on Dietary Reference Values for folate	For adults, the Average Requirement (AR) is determined from the folate intake required to maintain folate adequacy characterized	[6]

		by serum and red blood cell folate concentrations of ≥ 10 and 340 nmol/L, respectively	
Hesperidin	Scientific Opinion on the substantiation of a health claim related to a combination of diosmin, troxerutin and hesperidin and maintenance of normal venous tone pursuant to Article 13(5) of Regulation (EC) No 1924/2006,<br><br>Safety and efficacy of a feed additive consisting of an aqueous extract of Citrus limon (L.) Osbeck (lemon extract) for use in all animal species (Nor-Feed SAS).<br>Reevaluation of neohesperidine dihydrochalcone (E 959) as a food additive	A cause and effect relationship has not been established between the consumption of a combination of diosmin, troxerutin and hesperidin and the maintenance of normal venous tone.<br><br>No concerns for consumers were identified following the use of lemon extract up to the highest safe level in feed. The additive should be considered a skin and eye irritant, and a potential corrosive. The Panel concluded that dietary exposure to the food additive neohesperidine dihydrochalcone (E 959) at the reported uses and use levels would not raise a safety concern.	[7], [8], [9]
EGCG, (-)-epicatechin	Scientific opinion on the safety of green tea catechins	Based on the available data on the potential adverse effects of green tea catechins on the liver, the Panel concluded that there is evidence from interventional clinical trials that intake of doses equal or above 800 mg EGCG/day taken as a food supplement has been shown to induce a statistically significant increase of serum transaminasesin treated subjects compared to control.	[10]
Eicosapentaenoic acid, Docosahexaenoic Acids	Scientific Opinion on the Tolerable Upper Intake Level of eicosapentaenoic acid (EPA), docosahexaenoic acid (DHA) and docosapentaenoic acid (DPA)	Supplemental intakes of EPA and DHA combined at doses up to 5g/day, and supplemental intakes of EPA alone up to 1.8 g/day, do not raise safety concerns for adults. Dietary recommendations for EPA and DHA based on cardiovascular risk considerations for European	[11]

		adults are between 250 and 500 mg/day. Supplemental intakes of DHA alone up to about 1g/day do not raise safety concerns for the general population. No data are available for DPA when consumed alone. In the majority of the human studies considered, fish oils, also containing DPA in generally unknown (but relatively low) amounts, were the source of EPA and DHA.	
Linolenic acid	Scientific Opinion on the substantiation of a health claim related to alpha linolenic acid and contribution to brain and nerve tissue development pursuant to Article 14 of Regulation (EC) No 1924/2006	A cause and effect relationship has been established between the dietary intake of alpha-linolenic acid and contribution to brain and nerve tissue development	[12]
Naringenin	Assessment of the feed additive consisting of naringin for all animal species for the renewal of its authorisation (HealthTech Bio Actives, S.L.U. (HTBA))	The FEEDAP Panel cannot conclude on the possible respiratory sensitisation of the additive, due to the lack of data. There was no need for assessing the efficacy of the additive in the context of the renewal of the authorisation.	[2]
β-carotene	Statement on the safety of β-carotene use in heavy smokers	The Panel concluded that exposure to β-carotene from its use as food additive and as food supplement at a level below 15 mg/day do not give rise to concerns about adverse health effects in the general population, including heavy smokers.	[13]
Diosmetin	Scientific Opinion on the substantiation of a health claim related to a combination of diosmin, troxerutin and hesperidin and maintenance of normal venous tone pursuant to Article	A cause and effect relationship has not been established between the consumption of a combination of diosmin, troxerutin and hesperidin and the maintenance of normal venous tone. No concerns for consumers were identified following the use of lemon	[7], [8]

	13(5) of Regulation (EC) No 1924/2006,<br><br>Safety and efficacy of a feed additive consisting of a flavonoid-rich dried extract of Citrus × aurantium L. fruit (bitter orange extract) for use in all animal species (FEFANA asbl)	extract up to the highest safe level in feed.<br><br>The additive should be considered a skin and eye irritant, and a potential corrosive. The Panel concluded that dietary exposure to the food additive neohesperidinedihydrochalcone (E 959) at the reported uses and use levels would not raise a safety concern. The use in water for drinking is safe provided that the total daily intake of the additive does not exceed the daily amount that is considered safe when consumed via feed, except dog, cat and ornamental fish. No concerns for consumer safety were identified following the use of the additive up to the highest safe level in feed for the target animals.	
Hydroxytyrosol, Tyrosol	Safety of hydroxytyrosol as a novel food pursuant to Regulation (EC) No 258/97,<br><br>The EFSA Health Claim on Olive Oil Polyphenols: Acid Hydrolysis Validation and Total Hydroxytyrosol and Tyrosol Determination in Italian Virgin Olive Oils	The novel food, hydroxytyrosol, is safe under the proposed uses and use levels. The content of oleuropein and ligstroside derivatives in extra virgin olive oils (EVOOs) was indirectly evaluated comparing the number of phenols before and after hydrolysis. After acidic hydrolysis, a high content of total tyrosol was found in most of the EVOOs. The use of a suitable corrective factor for the evaluation of hydroxytyrosol allows an accurate determination only using pure tyrosol as a standard.	[14], [15]
Oleuropein	Scientific Opinion on the substantiation of a health claim related to olive (Olea europaea L.) leaf water extract and increase in glucose tolerance pursuant to Article 13(5) of Regulation (EC) No 1924/2006	The scientific evidence is insufficient to establish a cause and effect relationship between the consumption of olive leaf water extract and an increase in glucose tolerance.	[16]
Sulforaphane	Scientific Opinion Part I on the substantiation of	The claimed effects are “cardiovascular health”, “excellent source of	[17]

	health claims related to various food(s)/food constituent(s) not supported by pertinent human data (ID 411, 559, 1174, 1184, 1197, 1380, 1409, 1656, 1667, 1670, 1763, 1767, 1806, 1884, 1908, 1997, 2141, 2159, 2243, 2244, 2325, 2331, 2333, 2336, 2652, 2717, 2727, 2752, 2788, 2861, 2870, 2885, 2894, 3077, 3101, 3516, 3595, 3726, 4252, 4288, 4290, 4406, 4509, 4709) pursuant to Article 13(1) of Regulation (EC) No 1924/2006	sulforaphane known to help in the management of heart health", and "help restoration of myocardial tissue". The Panel assumes that the target population is the general population.<br>In the context of the proposed wordings and the clarifications provided by Member States, the Panel assumes that the claimed effects relate to the maintenance of normal cardiac function. The Panel considers that maintenance of normal cardiac function is a beneficial physiological effect.	

Table **S3.** Clinical studies investigating the effects of phytochemicals or formulations on bone health.

Component	Disorder	Type of Intervention	Intervention / treatment	Conclusions	ClinicalTrials.gov Identifier
Tart cherries (rich in phenolics and anthocyanins)	Osteoporosis, Postmenopausal Osteoporosis, Age Related	Randomized, double-blind crossover trial	Duration: 90 days<br>Participants: 33 healthy women aged 65 -80 years<br><br>Dietary Supplement: tart cherry juice concentrate is a commercial product provided by King Orchards (Central Lake, MI)<br><br>Group A: 240 ml per day for 45 days<br>Group B: 240ml twice daily for 45 days	Short-term supplementation with the higher dose of tart cherry juice decreased bone resorption from baseline without altering bone formation and turnover biomarkers in this cohort [18].	NCT04167150

			Wash out period and then exchange the arms' treatments		
Resveratrol	Obesity Inflammation Insulin Sensitivity Osteoporosis	Randomized, double-blind, Placebo-Controlled Trial	Duration: 16 weeks<br>Participants: 74 middle-aged obese men with MetS recruited from the general community, of which 66 completed all visits<br><br>Dietary Supplement: Resveratrol (RSV)<br><br>Group A: Placebo twice daily<br>Group B: RSVhigh 500mg twice daily<br>Group C: RSVlow 75mg twice daily	High-dose RSV supplementation positively affects bone, primarily by stimulating formation or mineralization [19].	NCT01412645
b-cryptoxanthin plus phytosterols	Hypercholesterolemia Osteoporosis	Randomized, double-blind crossover trial	Duration: 12 weeks<br>Participants: 38 postmenopausal women<br><br>Group A: b-cryptoxanthin plus phytosterols<br>Group B: placebo	The intake of this beverage could contribute to reduce the risk of cardiovascular diseases also obtaining a beneficial effect on serum inflammatory status in postmenopausal women [20].	NCT02065024
Dried Plum (phenolic compounds)	Postmenopausal Osteoporosis	Randomized controlled trial	Duration: 52 weeks<br>Participants: 332<br><br>Group A: Dried Plum (50 or 100mg)<br>Group B: Calcium supplement<br>Group C: Vitamin D supplement	A 50 g dose of daily dose of prunes can prevent loss of total hip BMD in postmenopausal women, without increased fat mass seen with	NCT02822378

				the larger dose [21].	
Blueberry The hypothesis is that the polyphenolics found in blueberries will reduce calcium loss from bones.	Osteoporosis, Postmenopausal Bone Loss, Age-related	Randomized crossover trial	Duration: 52 weeks<br>Participants: 20 healthy women, aged 45 to 70 years<br><br>Group A: Blueberry Baseline<br>Group B: Blueberry Low (0,75 cups)<br>Group C: Blueberry Medium (1,5 cups)<br>Group D: Blueberry High (3 cups)	Not yet available	NCT02630797
Green tea extract	Osteoporosis	Randomized controlled trial	Duration: 24 weeks<br>Participants: 171 postmenopausal women with osteopenia<br><br>Group A: Placebo<br>Group B: Green Tea Polyphenols (GTP)<br>Group C: Placebo+Tai Chi (TC)<br>Group D: GTP+TC	GTP at a dose of 500 mg/day and/or TC exercise at 3 hr/week for 24 weeks appear to be safe in postmenopausal osteopenic women, particularly in terms of liver and kidney functions [22].	NCT00625391
Combined extract of mulberry and Vietnamese coriander (MP)	Healthy	Randomized, double-blind crossover trial	Duration: 8 weeks<br>Participants: 45 healthy perimenopausal and postmenopausal women<br><br>Group A: Placebo<br>Group B: MP 50	This study clearly revealed that subjects in Group C, increased the total phenolic compounds in serum and improved bone	NCT02562274

			mg/day<br>Group C: MP 1500 mg/day	formation markers including osteocalcin and alkaline phosphatase (ALP) but decreased bone resorption marker including serum collagen type 1 cross-linked C-telopeptide (beta CTx) [23].	
Fermented red clover (RC) extract (Rich in isoflavone aglycones and probiotics)	Osteopenia<br>Osteoporosis	Randomized controlled trial	Duration: 8 weeks<br>Participants: 45 healthy perimenopausal and postmenopausal women<br><br>Group A: Daily Red clover extract 80mg, Ca 1.040mg, VitD 25mg and Mg 487mg<br>Group B: Daily Ca 1.040mg, VitD 25mg and Mg 487mg	Twice daily RCE intake over 1 year potently attenuated BMD loss caused by estrogen deficiency, improved bone turnover, promoted a favorable estrogen metabolite profile (2-OH:16α-OH), and stimulated equol production in postmenopausal women with osteopenia. RCE intake combined with supplementation (calcium, magnesium, and calcitriol) was more effective than supplementation alone [24].	NCT02174666
Mediterranean Diet	Osteoporosis	Controlled trial	Duration: 24 weeks	Not available yet	NCT01653275

	Postmenopausal Bone Loss		Participants: 22 postmenopausal women<br><br>Mediterranean Style Diet (olive oil, walnuts, frozen portions of high n-3 LCPUFA fish)		
Diet with increased fruits, vegetables, and calcium	Osteoporosis	Randomized Controlled trial	Duration: 2 years<br>Participants: 228 girls 14- to 16-year-old with mass index below the national median<br><br>Behavioral: Diet with increased fruits, vegetables, and calcium<br>Behavioral: Increased high impact activity and resistance training	A comprehensive health care–based lifestyle intervention can effectively improve dietary intake and increase bone mineral gains in adolescent girls [25].	NCT00067600
Genistein (soy isoflavones)	Osteopenia<br><br>Osteoporosis	Blinded randomized crossover trial	Duration: > 4 years<br>Participants: 24 healthy postmenopausal women<br><br>Group A: 5 soy isoflavone oral supplements (2 doses of a genistein-rich soy supplement and 3 doses of mixed isoflavones in various proportions)<br>Group B: risedronate (a bisphosphonate)	Soy isoflavones, although not as potent as risedronate, are effective bone-preserving agents in postmenopausal women regardless of their equol-producing status, and mixed isoflavones in their natural ratios are more effective than	NCT00244907

				enriched genistein [26].	
Hesperidin (flavonoid)	Osteopenia<br><br>Osteoporosis	Double blinded<br><br>Randomized controlled trial	Duration: > 4 years<br>Participants: 110 healthy 50-65 years old<br><br>Group A: Hesperidin<br>Group B: Placebo	Not available yet	NCT00330096
Soy isoflavones	Osteoporosis	Double blinded<br><br>Randomized controlled trial	Duration: 2 years<br>Participants: 403 healthy women 40 – 60 years old<br><br>Group A: Soy isoflavones (80mg)<br>Group B: Soy isoflavones (120mg)<br>Group C: Placebo	Daily supplementation for 2 years with 80–120 mg soy hypocotyl isoflavones has minimal risk in healthy menopausal women [27].	NCT00665860
Isoflavones-enriched foods	Osteoporosis	Double blinded<br><br>Randomized controlled trial	Duration: 1 year<br>Participants: 300 post-menopausal healthy women 40 – 65 years old<br><br>Group A: Isoflavones-enriched foods<br>Group B: Placebo	Consumption of foods containing 110 mg/day of soy isoflavone aglycone equivalents for 1 year did not prevent postmenopausal bone loss and did not affect bone turnover in apparently healthy early postmenopausal white women [28].	NCT00301353

2.2. MD simulations

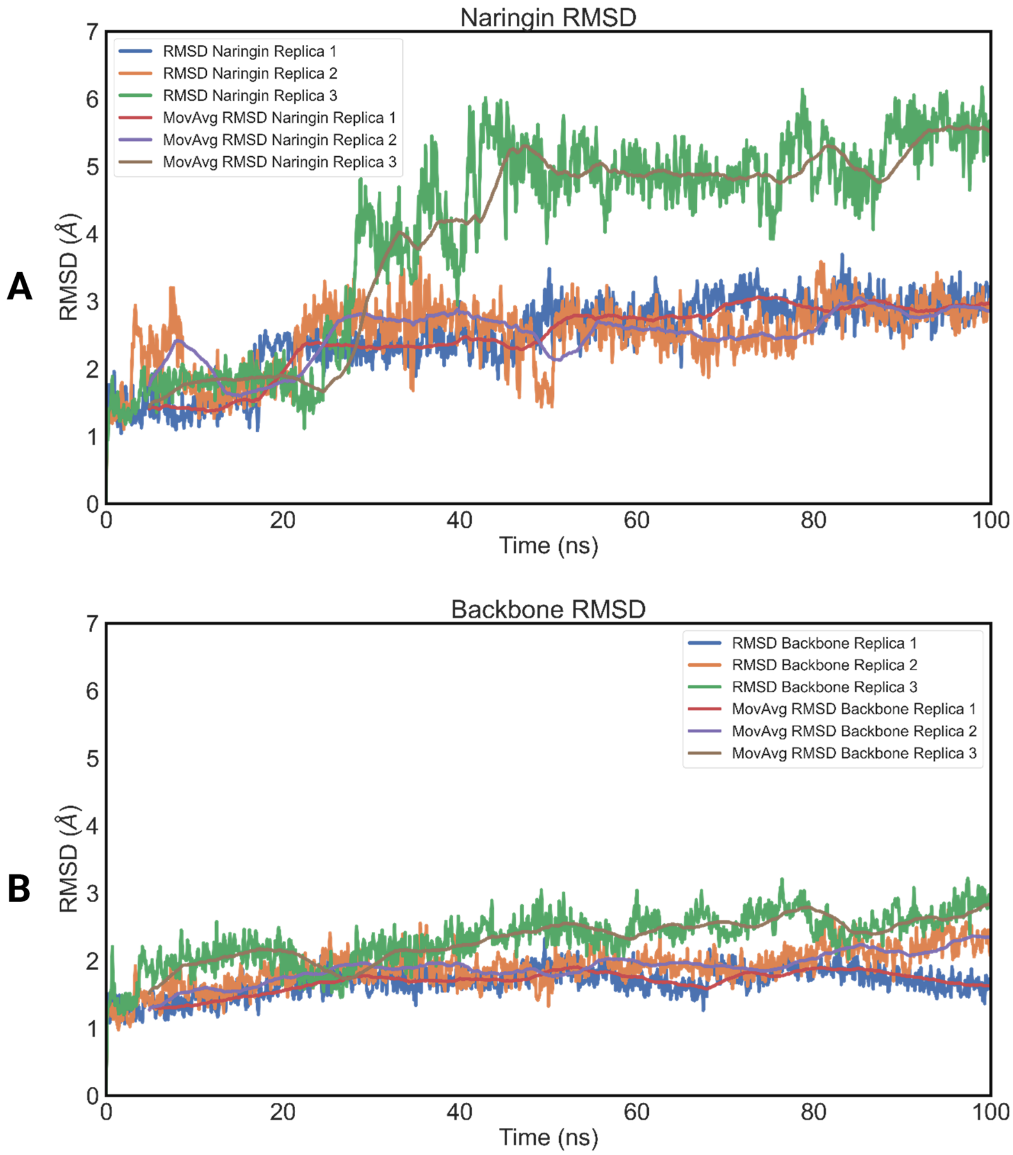

Figure S1. A) RMSD of the three replicas of naringin bound to ERK1 as a function of time, B) RMSD of the three replicas of the protein backbone of ERK1 as a function of time.

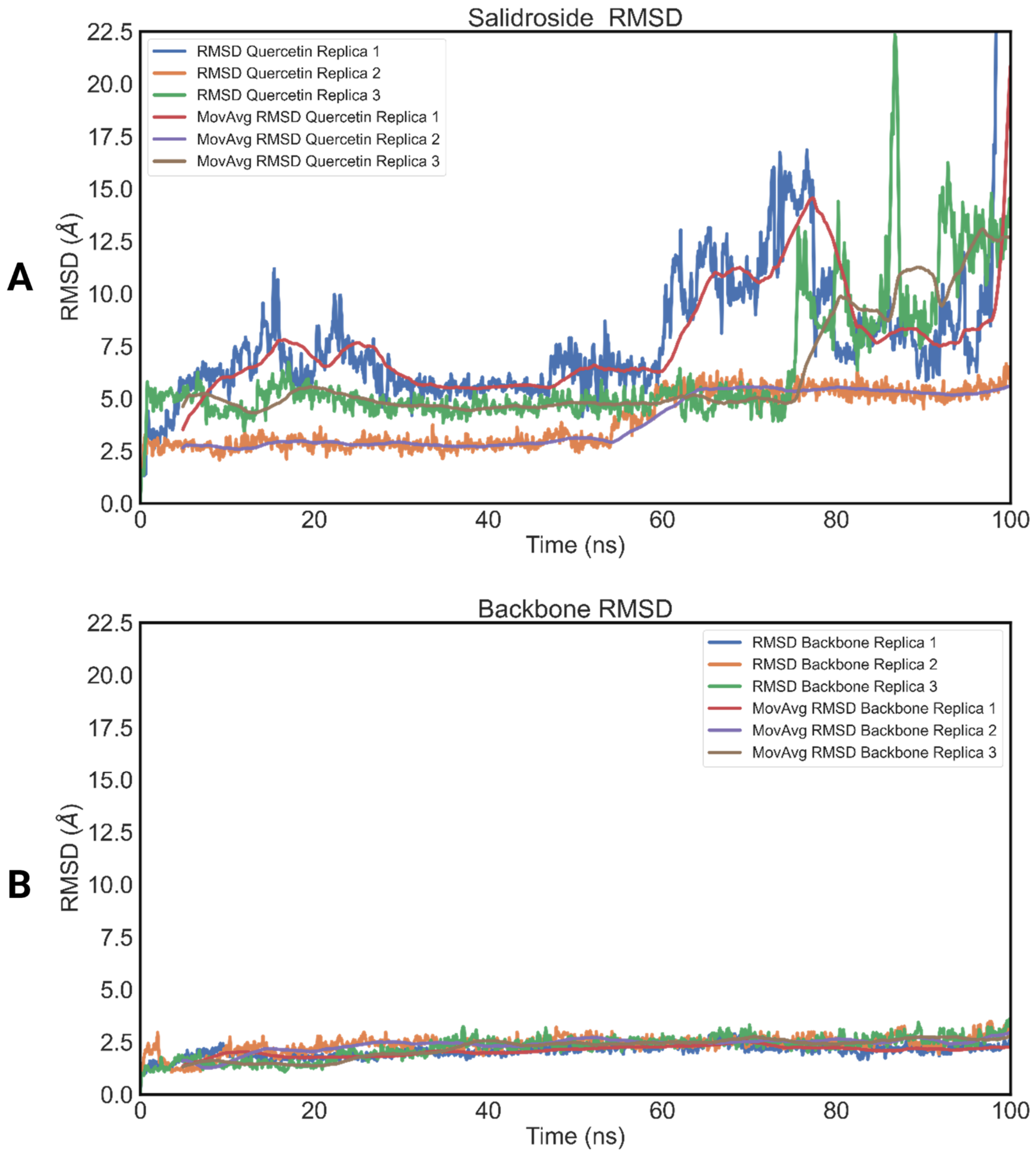

Figure S2. A) RMSD of the three replicas of salidroside bound to ERK1 as a function of time, B) RMSD of the three replicas of the protein backbone of ERK1 as a function of time.

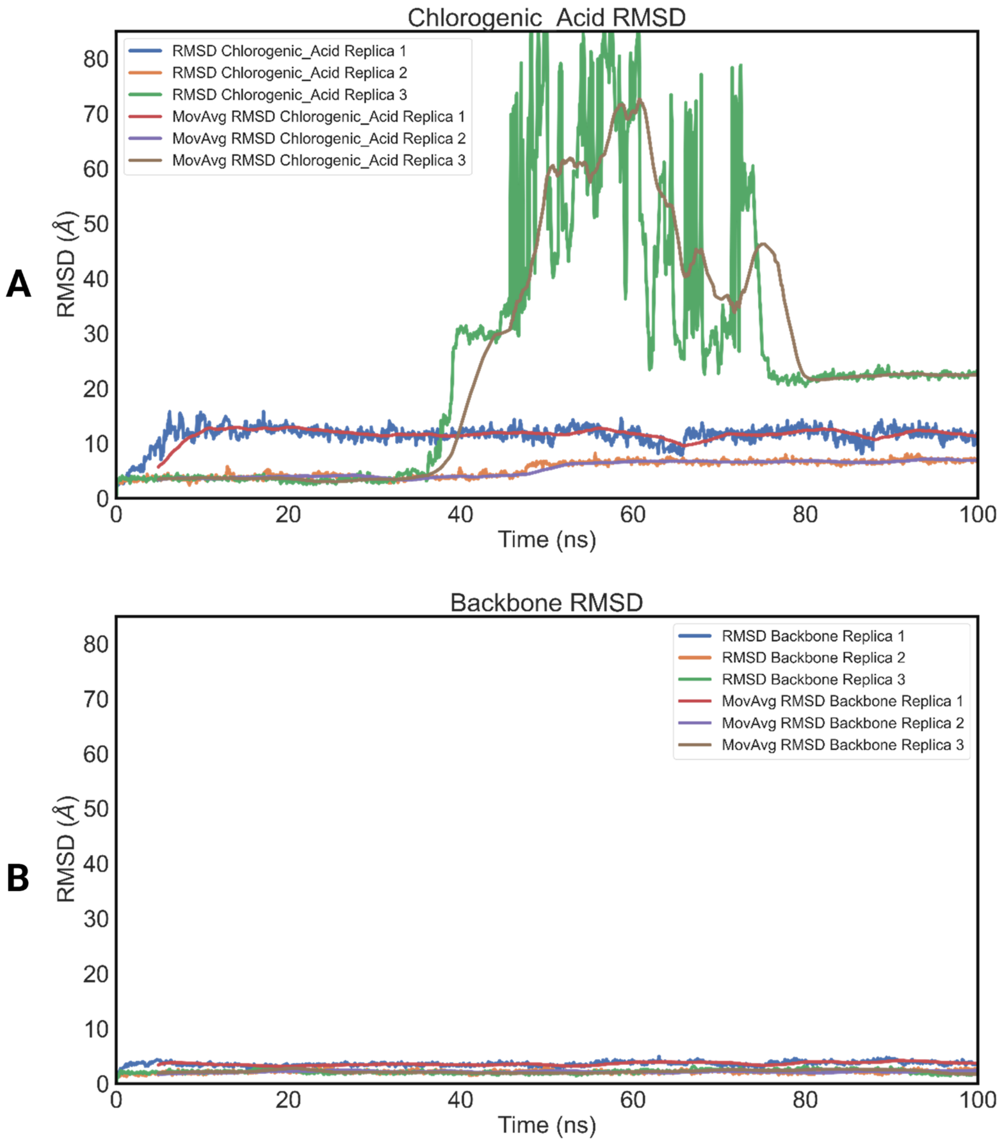

Figure S3. A) RMSD of the three replicas of chlorogenic acid bound to ERK1 as a function of time, B) RMSD of the three replicas of the protein backbone of ERK1 as a function of time.

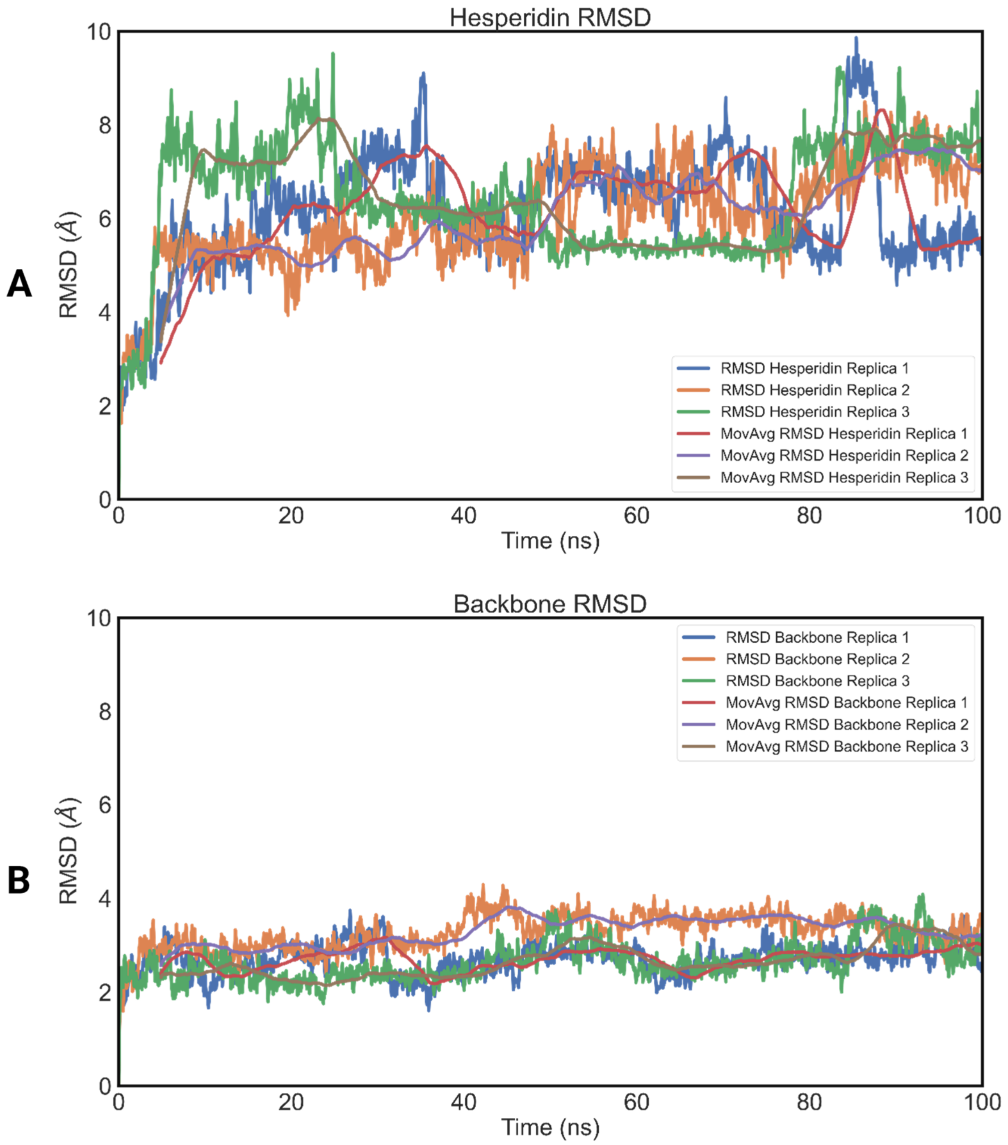

Figure S4. A) RMSD of the three replicas of hesperidin bound to ERK1 as a function of time, B) RMSD of the three replicas of the protein backbone of ERK1 as a function of time.

Table S4. Average values of RMSD and standard deviation of the protein backbone for each ERK1-ligand complex were calculated over the 100 ns MD simulations.

Replica ID	RMSD quercetin (Å)	RMSD naringin (Å)	RMSD salidroside (Å)	RMSD chlorogenic acid (Å)	RMSD hesperidin (Å)
1	1.53 ± 0.19	1.70 ± 0.24	2.07 ± 0.30	3.63 ± 0.40	2.70 ± 0.34
2	1.71 ± 0.30	1.90 ± 0.29	2.39 ± 0.41	2.17 ± 0.28	3.28 ± 0.39
3	1.81 ± 0.26	2.32 ± 0.38	2.23 ± 0.53	2.22 ± 0.35	2.65 ± 0.41

Average RMSD	1.68 ± 0.13	1.97 ± 0.32	2.23 ± 0.16	2.67 ± 0.82	2.87 ± 0.35

A

B

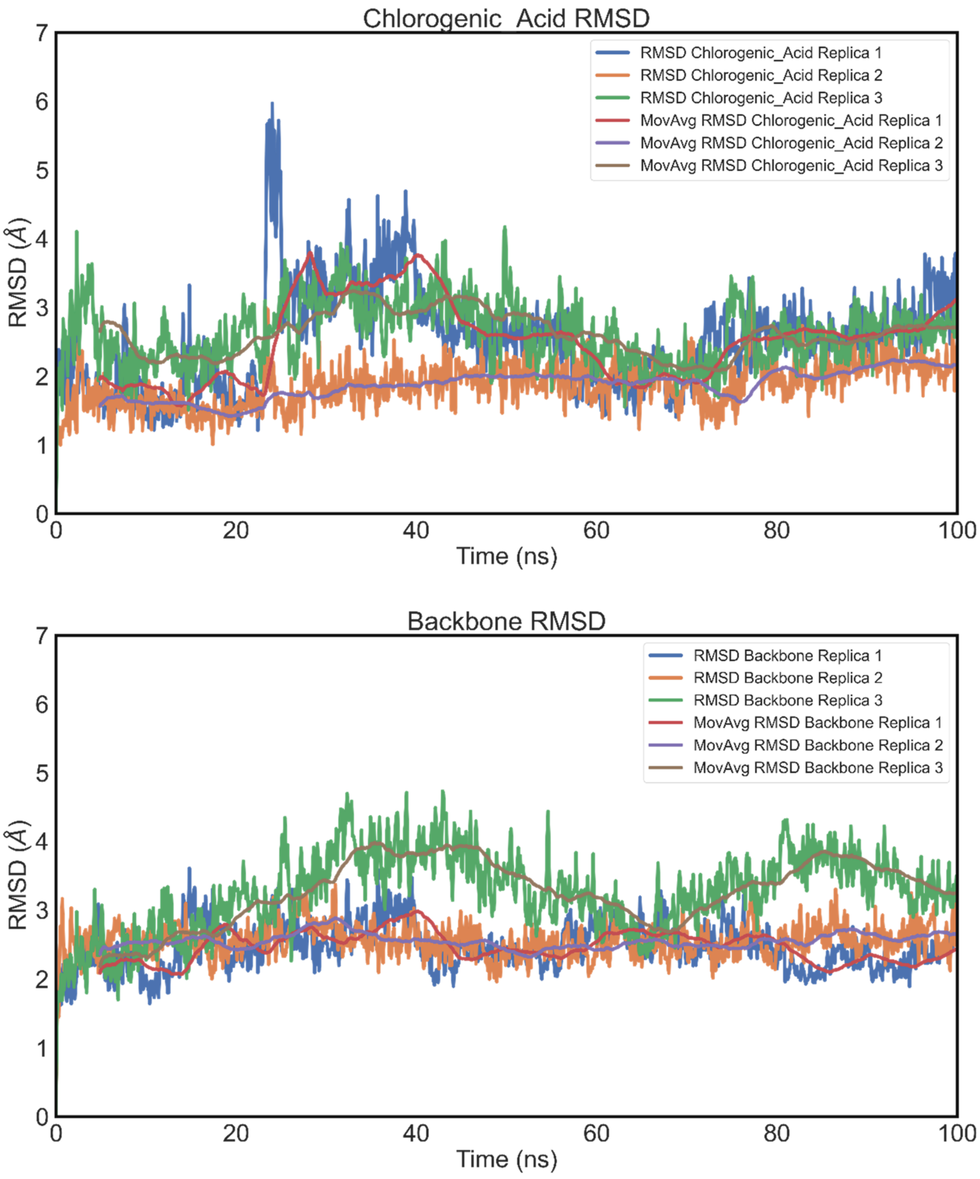

Figure S5. A) RMSD of the three replicas of chlorogenic acid bound to p38b as a function of time, B) RMSD of the three replicas of the protein backbone of p38b as a function of time.

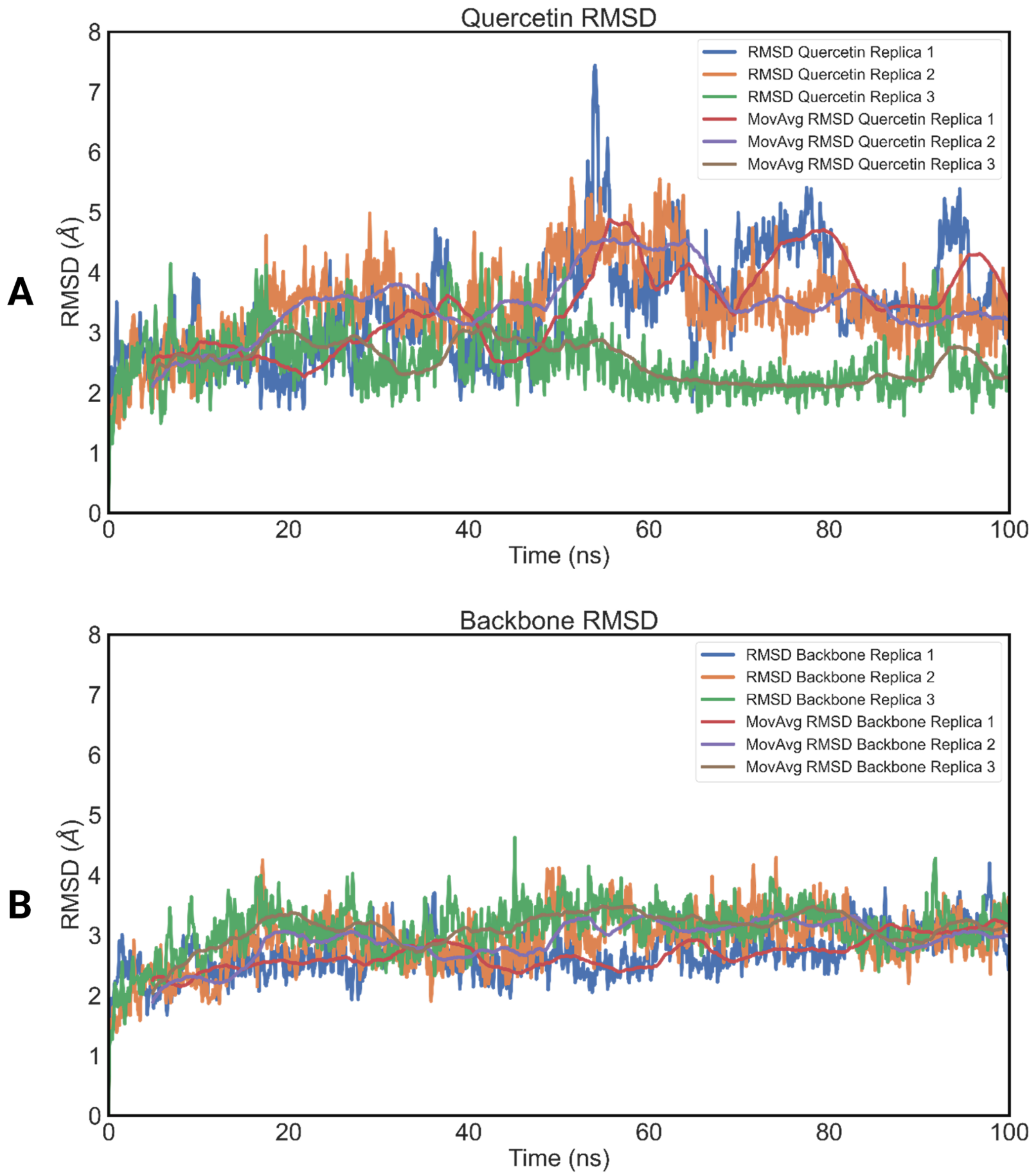

Figure S6. A) RMSD of the three replicas of quercetin bound to p38b as a function of time, B) RMSD of the three replicas of the protein backbone of p38b as a function of time.

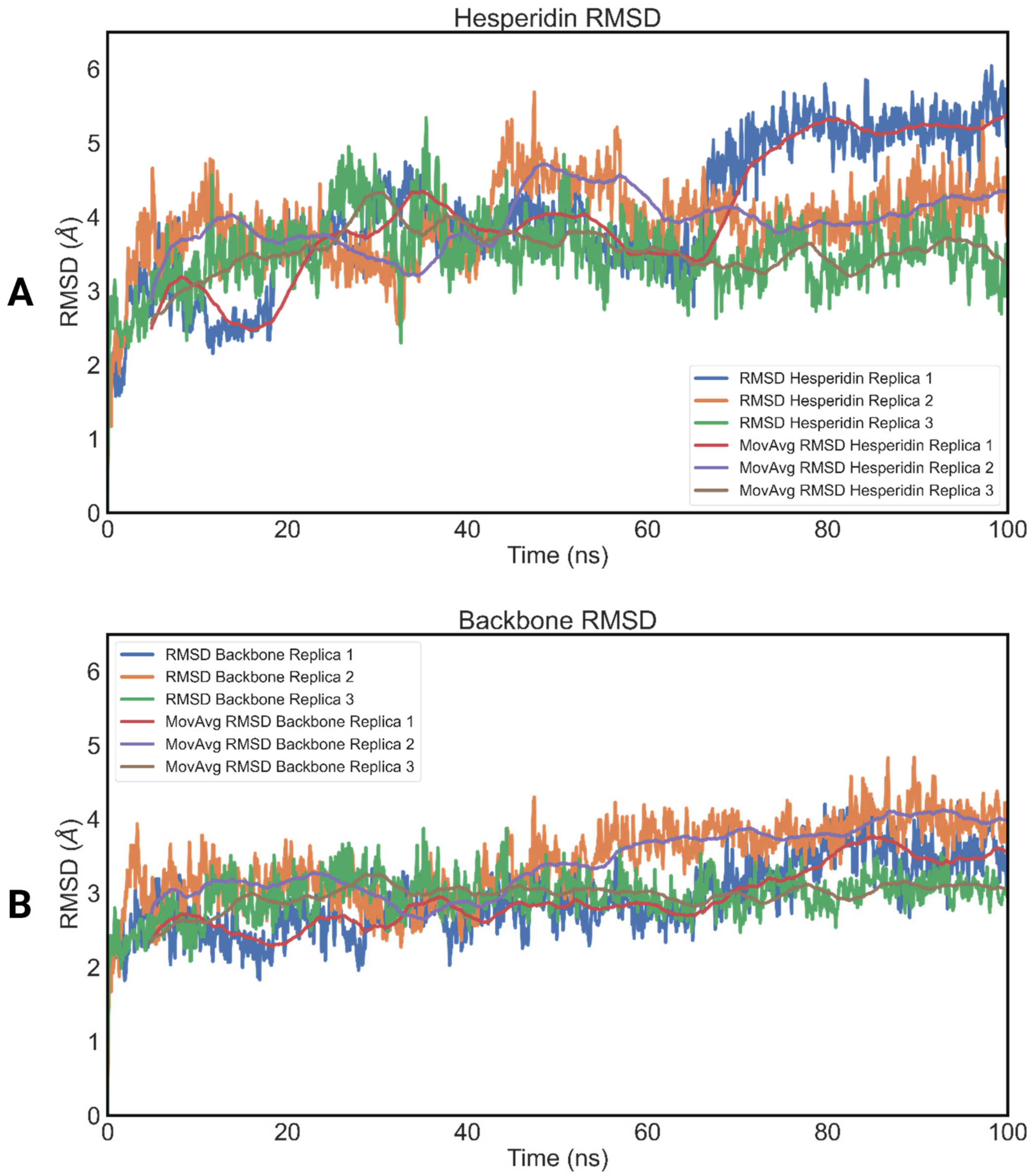

Figure S7. A) RMSD of the three replicas of hesperidin bound to p38b as a function of time, B) RMSD of the three replicas of the protein backbone of p38b as a function of time.

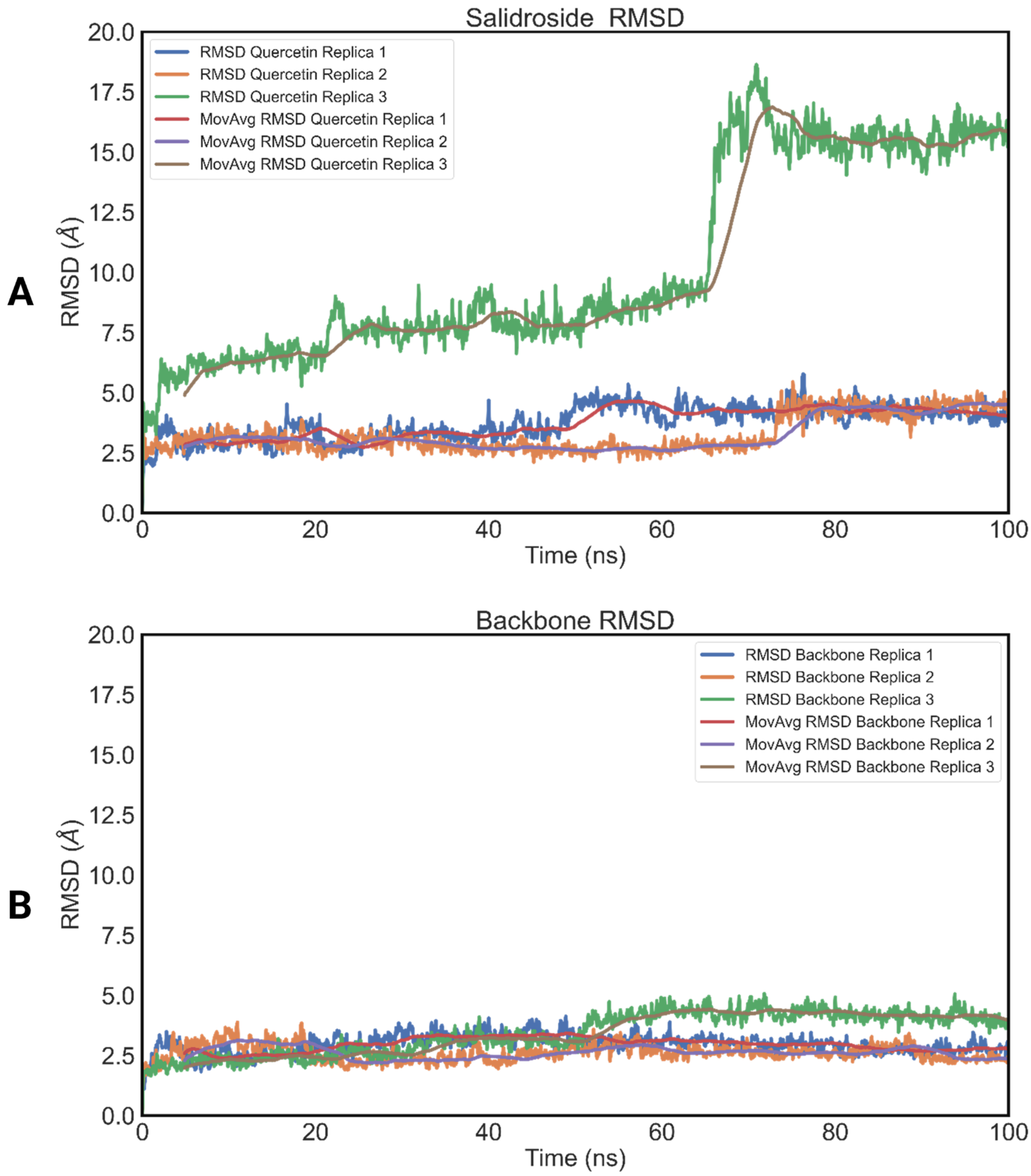

Figure S8. A) RMSD of the three replicas of salidroside bound to p38b as a function of time, B) RMSD of the three replicas of the protein backbone of p38b as a function of time.

Table S5. Average values of RMSD and standard deviation of the protein backbone for each p38b-ligand complex were calculated over the 100 ns MD simulations.

Replica ID	RMSD quercetin (Å)	RMSD naringin (Å)	RMSD salidroside (Å)	RMSD chlorogenic acid (Å)	RMSD hesperidin (Å)
1	2.69 ± 0.37	2.84 ± 0.39	2.95 ± 0.40	2.45 ± 0.34	2.93 ± 0.49
2	2.89 ± 0.47	2.83 ± 0.47	2.60 ± 0.36	2.55 ± 0.25	3.42 ± 0.53
3	3.10 ± 0.42	2.59 ± 0.27	3.42 ± 0.86	3.24 ± 0.58	2.96 ± 0.29

Average RMSD	2.89 ± 0.20	2.76 ± 0.14	2.99 ± 0.41	2.75 ± 0.43	3.10 ± 0.28

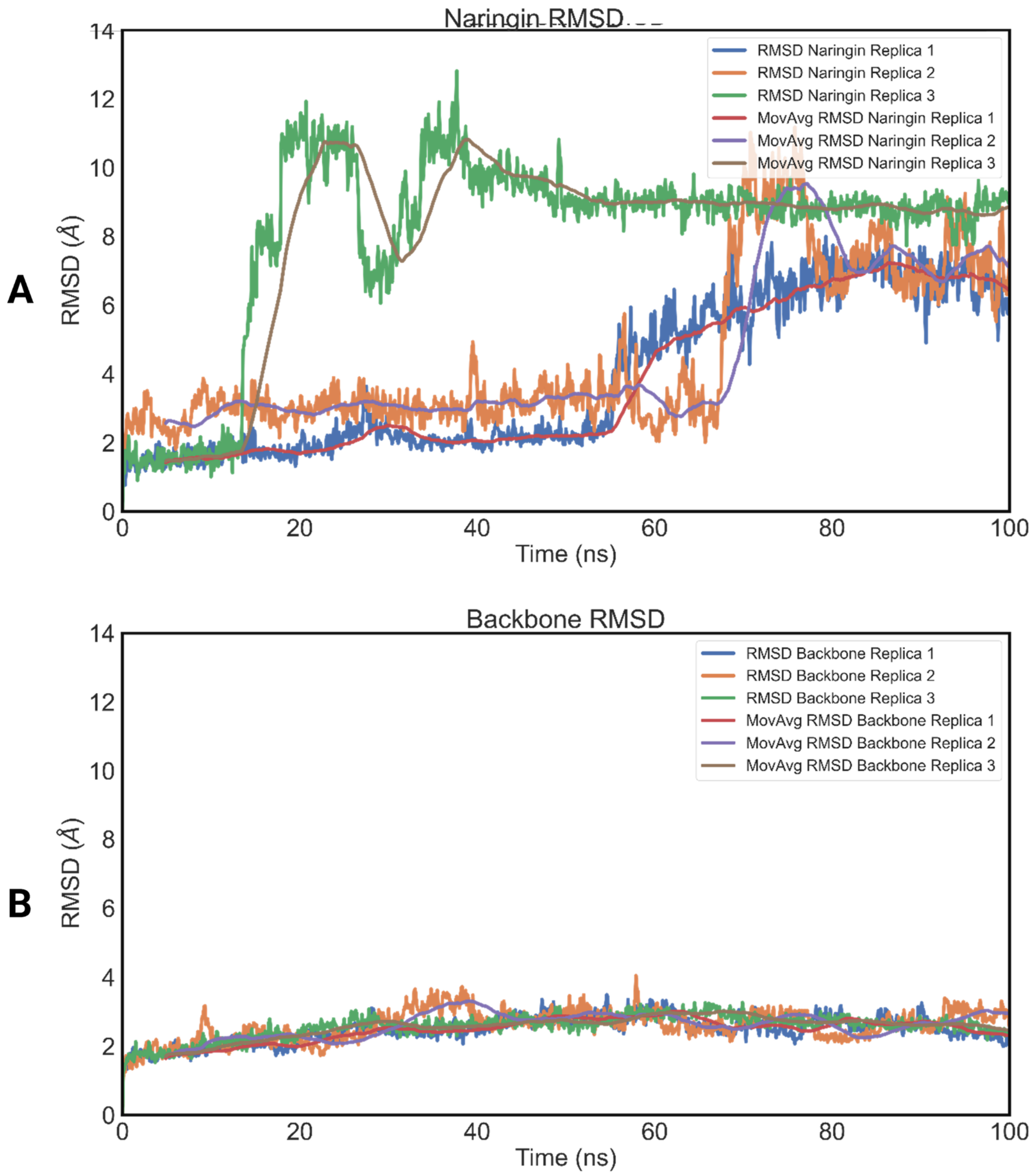

Figure S9. A) RMSD of the three replicas of naringin bound to JNK1 as a function of time, B) RMSD of the three replicas of the protein backbone of JNK1 as a function of time.

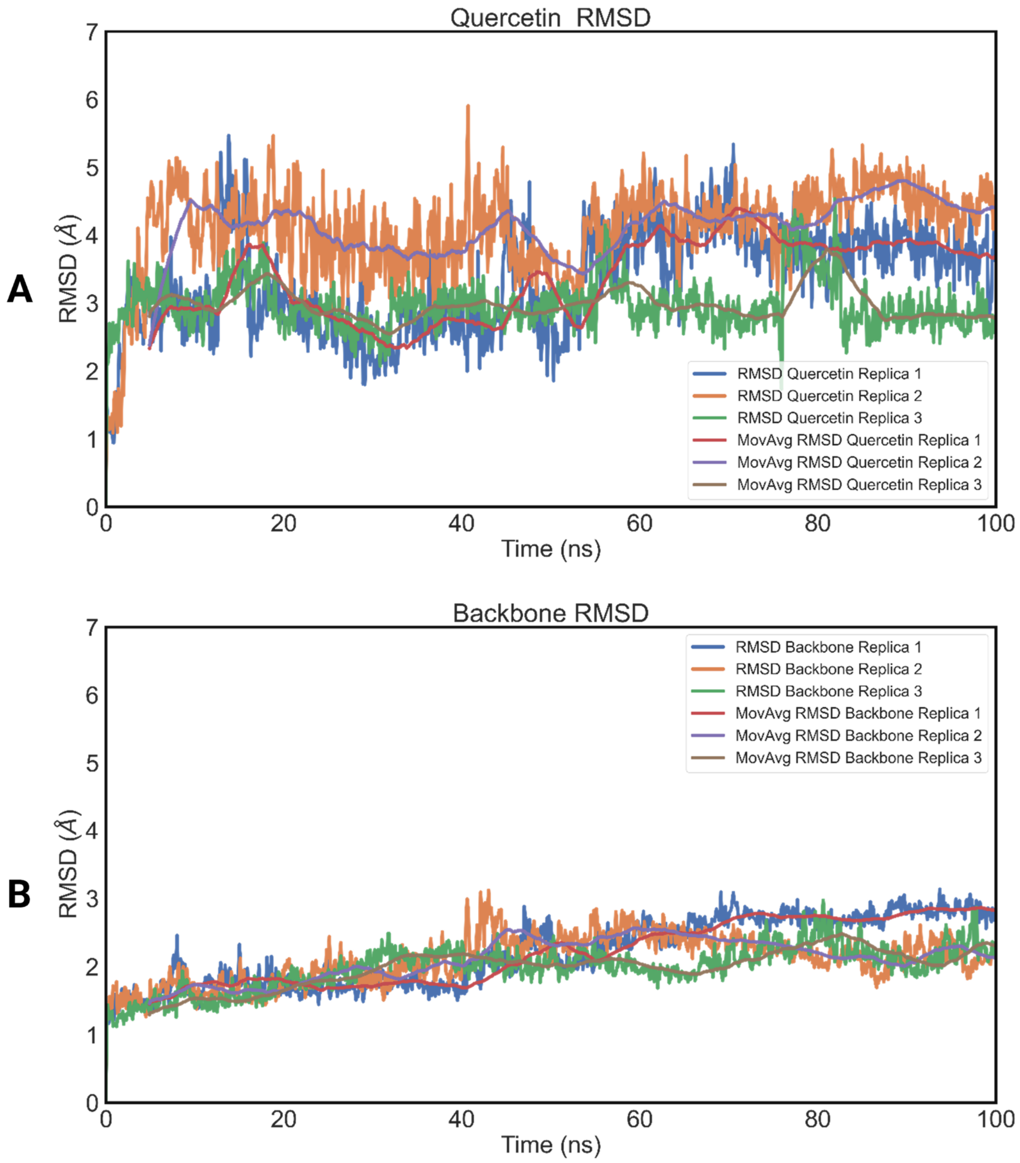

Figure S10. A) RMSD of the three replicas of quercetin bound to JNK1 as a function of time, B) RMSD of the three replicas of the protein backbone of JNK1 as a function of time.

Table S6. Average values of RMSD and standard deviation of each JNK1-ligand complex were calculated over the 100 ns MD simulations.

Replica ID	RMSD quercetin (Å)	RMSD naringin (Å)
1	3.34 ± 0.77	3.88 ± 2.23
2	4.09 ± 0.67	4.58 ± 2.33
3	2.97 ± 0.37	8.08 ± 2.75
Average RMSD	3.47 ± 0.56	5.51 ± 2.25

Table S7. Average values of RMSD and standard deviation of the protein backbone for each JNK1-ligand complex were calculated over the 100 ns MD simulations.

Replica ID	RMSD quercetin (Å)	RMSD naringin (Å)
1	2.21 ± 0.49	2.46 ± 0.39
2	2.10 ± 0.35	2.60 ± 0.45
3	1.98 ± 0.33	2.56 ± 0.35
Average RMSD	2.10 ± 0.11	2.54 ± 0.07

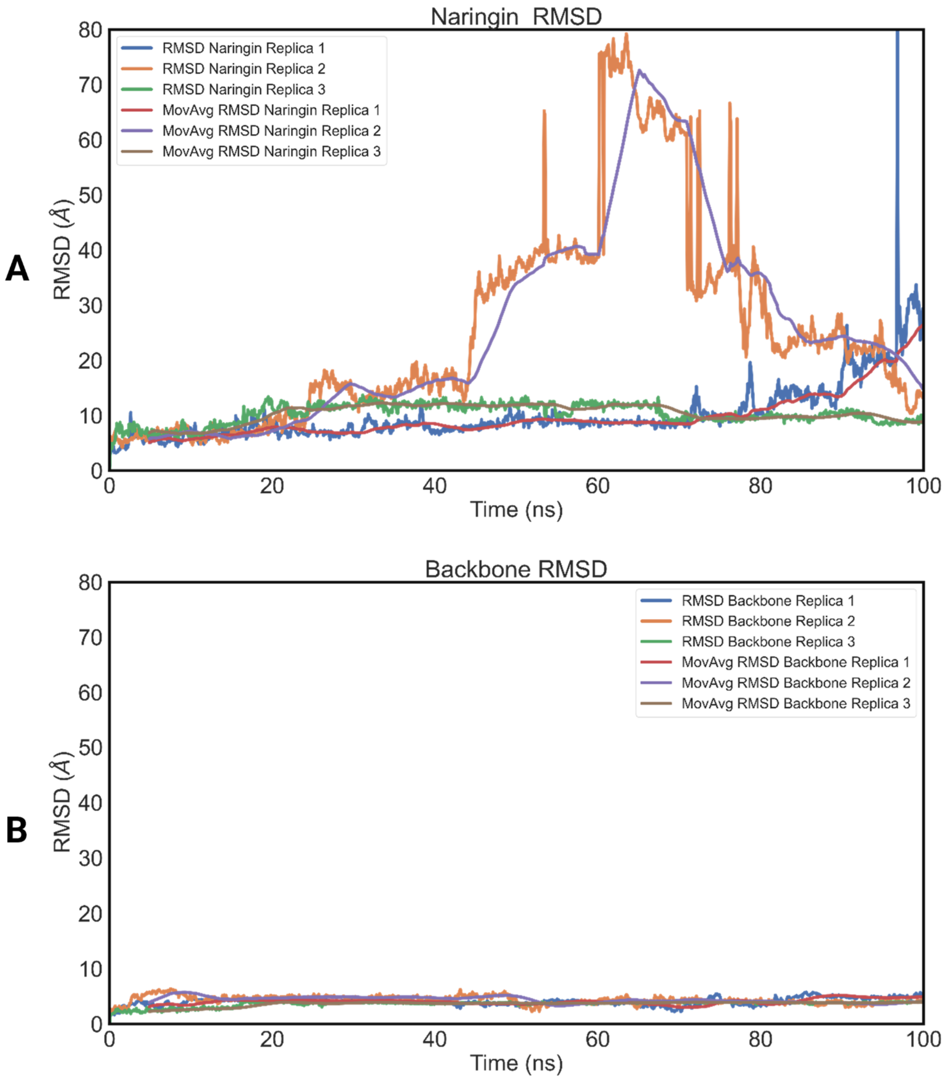

Figure S11. A) RMSD of the three replicas of naringin bound to p38c as a function of time, B) RMSD of the three replicas of the protein backbone of p38c as a function of time.

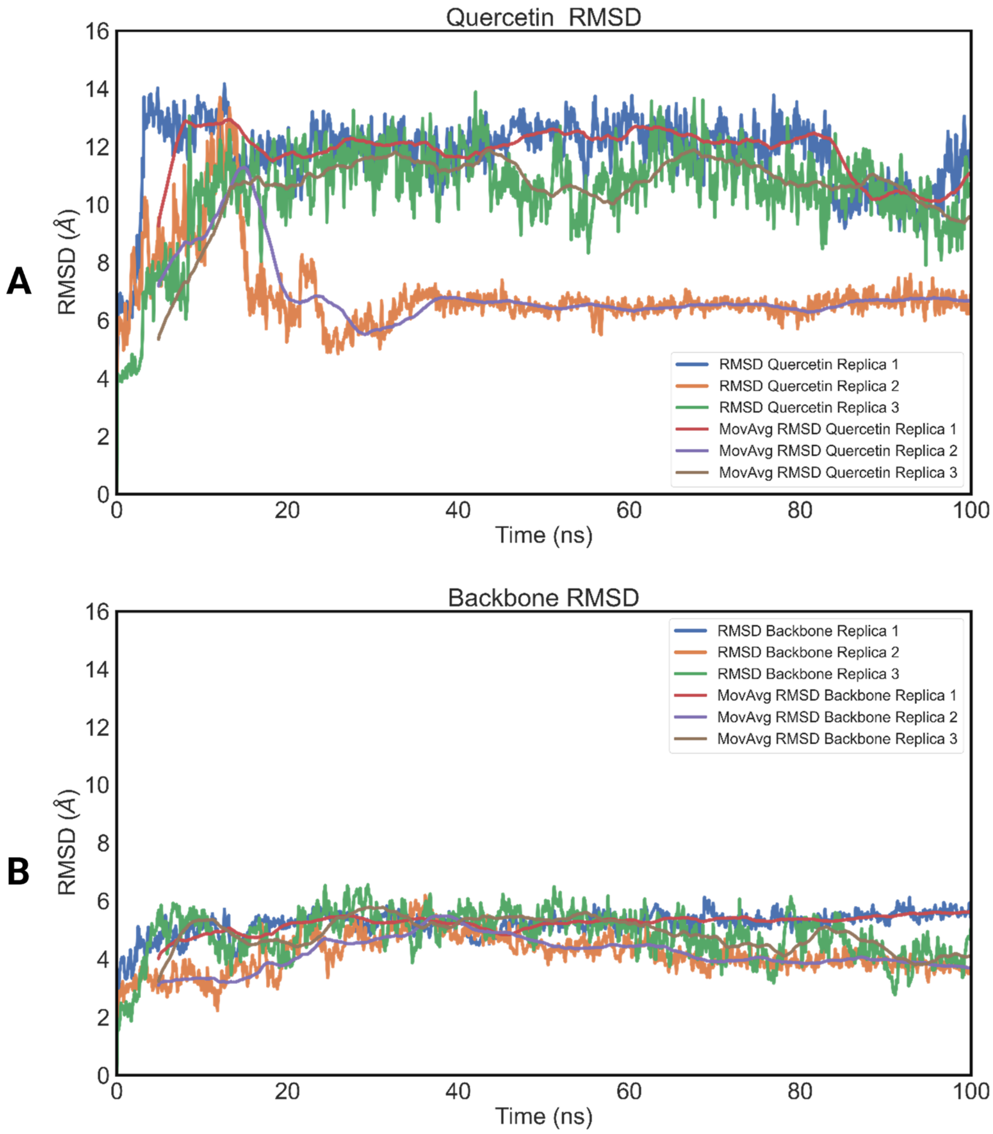

Figure S12. A) RMSD of the three replicas of quercetin bound to p38c as a function of time, B) RMSD of the three replicas of the protein backbone of p38c as a function of time.

Table S8. Average values of RMSD and standard deviation of each p38c-ligand complex were calculated over the 100 ns MD simulations.

Replica ID	RMSD quercetin (Å)	RMSD naringin (Å)
1	11.77 ± 1.32	10.08 ± 5.50
2	6.89 ± 1.31	25.43 ± 18.71
3	10.49 ± 1.70	10.28 ± 1.87
Average RMSD	9.72 ± 2.07	15.26 ± 7.19

Table S9. Average values of RMSD and standard deviation of the protein backbone for each p38c-ligand complex were calculated over the 100 ns MD simulations.

Replica ID	RMSD quercetin (Å)	RMSD naringin (Å)
1	5.22 ± 0.46	4.05 ± 0.66
2	4.16 ± 0.67	4.38 ± 0.72
3	4.83 ± 0.83	3.65 ± 0.50
Average RMSD	4.73 ± 0.53	4.02 ± 0.37

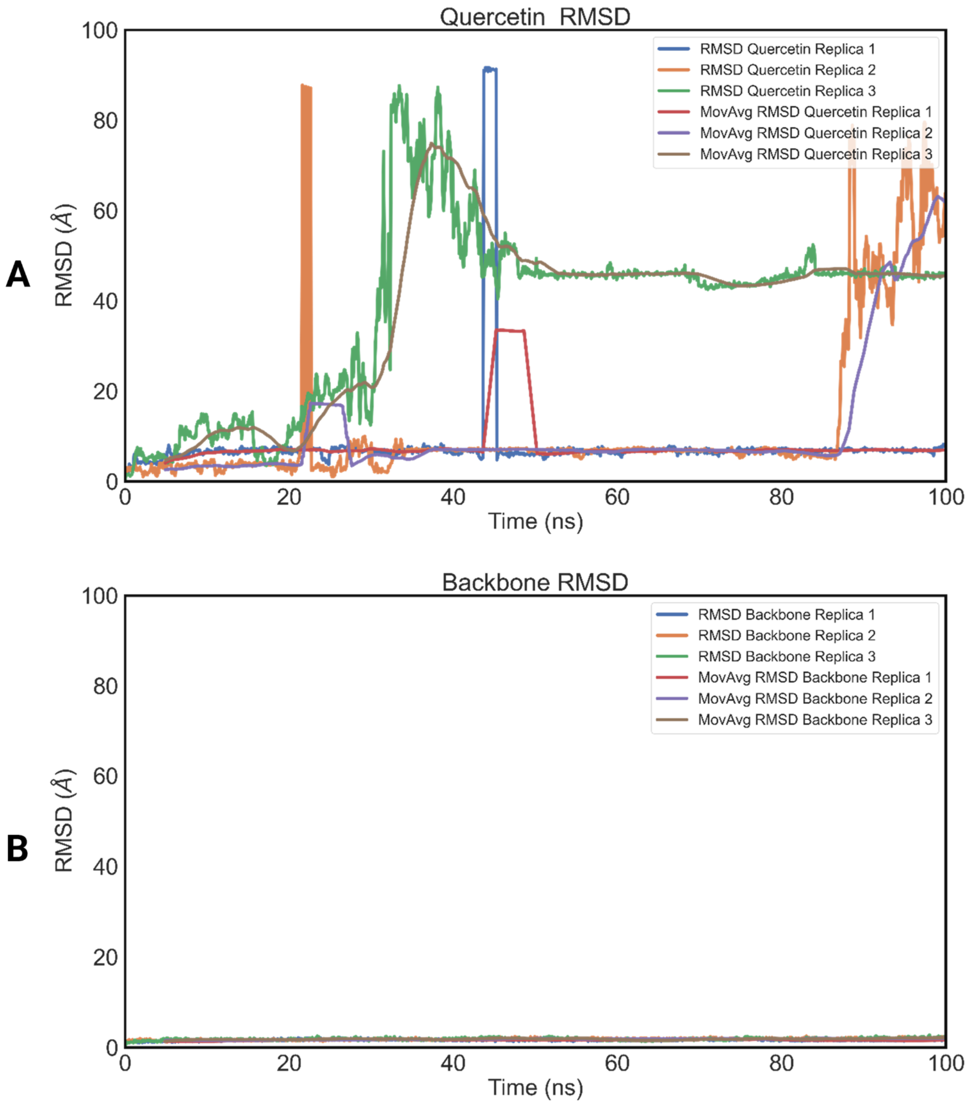

Figure S13. A) RMSD of the three replicas of quercetin bound to ERK2 as a function of time, B) RMSD of the three replicas of the protein backbone of p38c as a function of time.

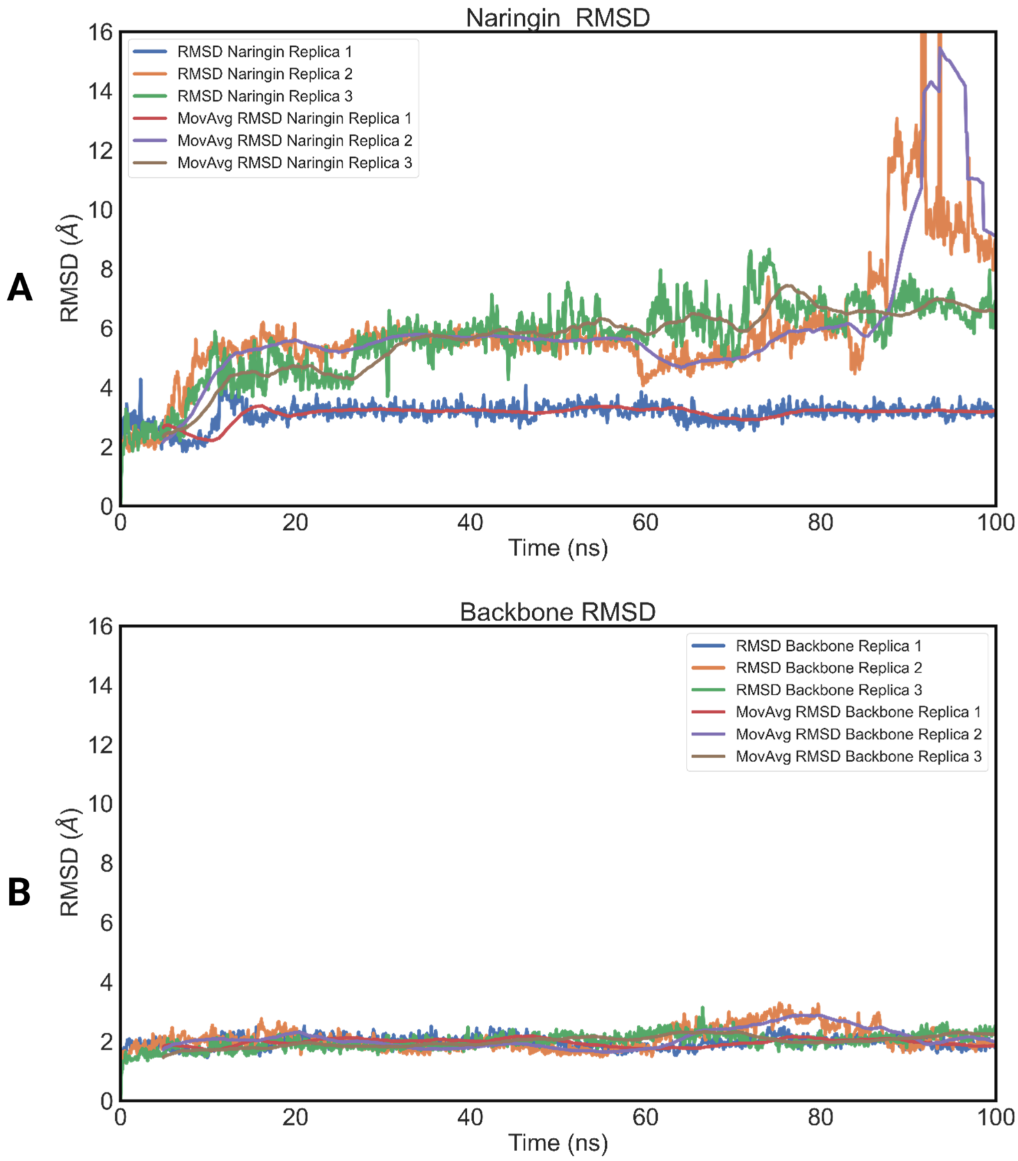

Figure S14. A) RMSD of the three replicas of naringin bound to ERK2 as a function of time, B) RMSD of the three replicas of the protein backbone of ERK2 as a function of time.

Table S10. Average values of RMSD and standard deviation of each ERK2-ligand complex were calculated over the 100 ns MD simulations.

Replica ID	RMSD quercetin (Å)	RMSD naringin (Å)
1	7.99 ± 10.65	3.12 ± 0.36
2	12.44 ± 17.97	6.10 ± 4.77
3	38.00 ± 19.45	5.61 ± 1.32
Average RMSD	19.48 ± 13.22	4.94 ± 1.30

Table S11. Average values of RMSD and standard deviation of the protein backbone for each ERK2-ligand complex were calculated over the 100 ns MD simulations.

Replica ID	RMSD quercetin (Å)	RMSD naringin (Å)
1	1.68 ± 0.20	1.96 ± 0.20
2	1.88 ± 0.22	2.08 ± 0.38
3	1.90 ± 0.26	2.00 ± 0.26
Average RMSD	1.82 ± 0.12	2.01 ± 0.06

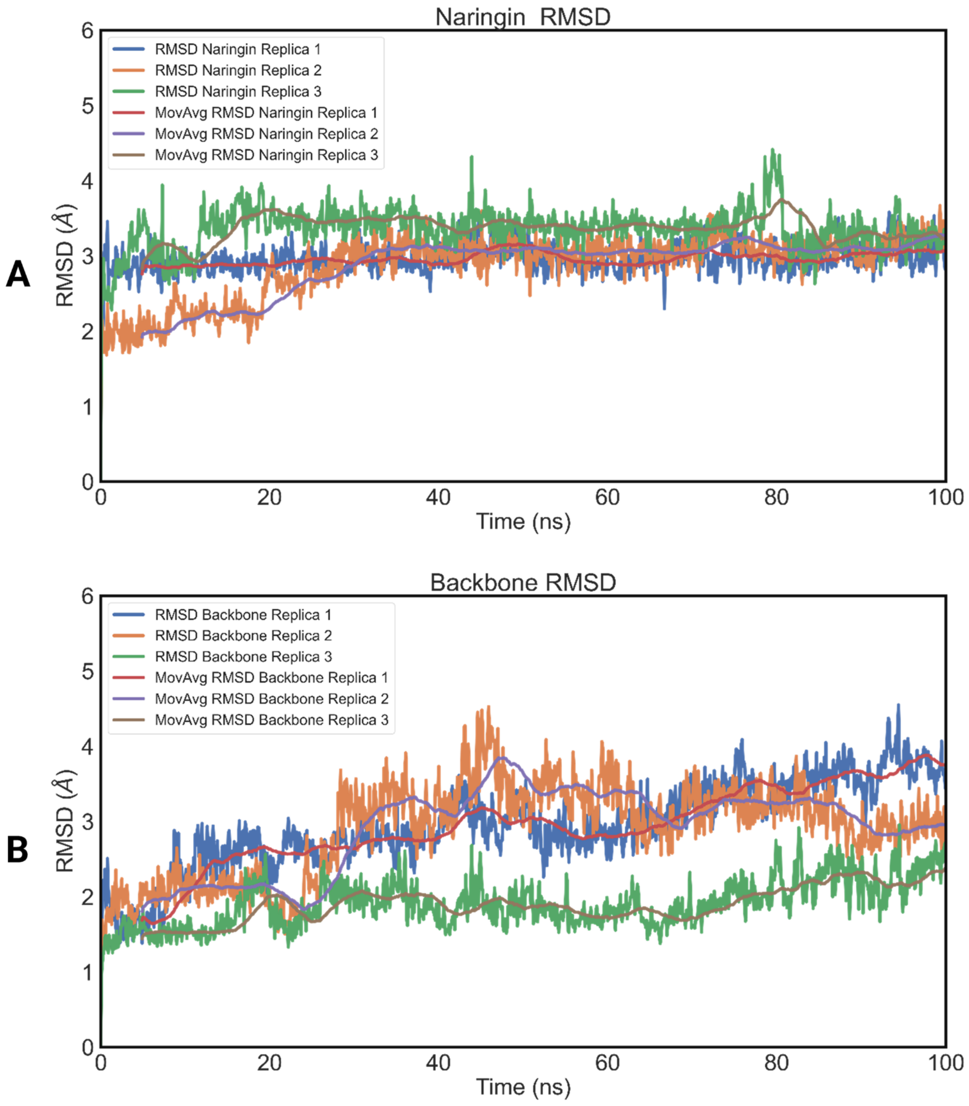

Figure S15. A) RMSD of the three replicas of naringin bound to JNK2 as a function of time, B) RMSD of the three replicas of the protein backbone of JNK2 as a function of time.

Table S12. Average values of RMSD and standard deviation of the protein backbone for each JNK2-ligand complex were calculated over the 100 ns MD simulations.

Replica ID	RMSD quercetin (Å)	RMSD naringin (Å)
1	2.36 ± 0.41	2.95 ± 0.58
2	1.90 ± 0.24	2.90 ± 0.62
3	1.95 ± 0.25	1.90 ± 0.32
Average RMSD	2.07 ± 0.25	2.58 ± 0.59

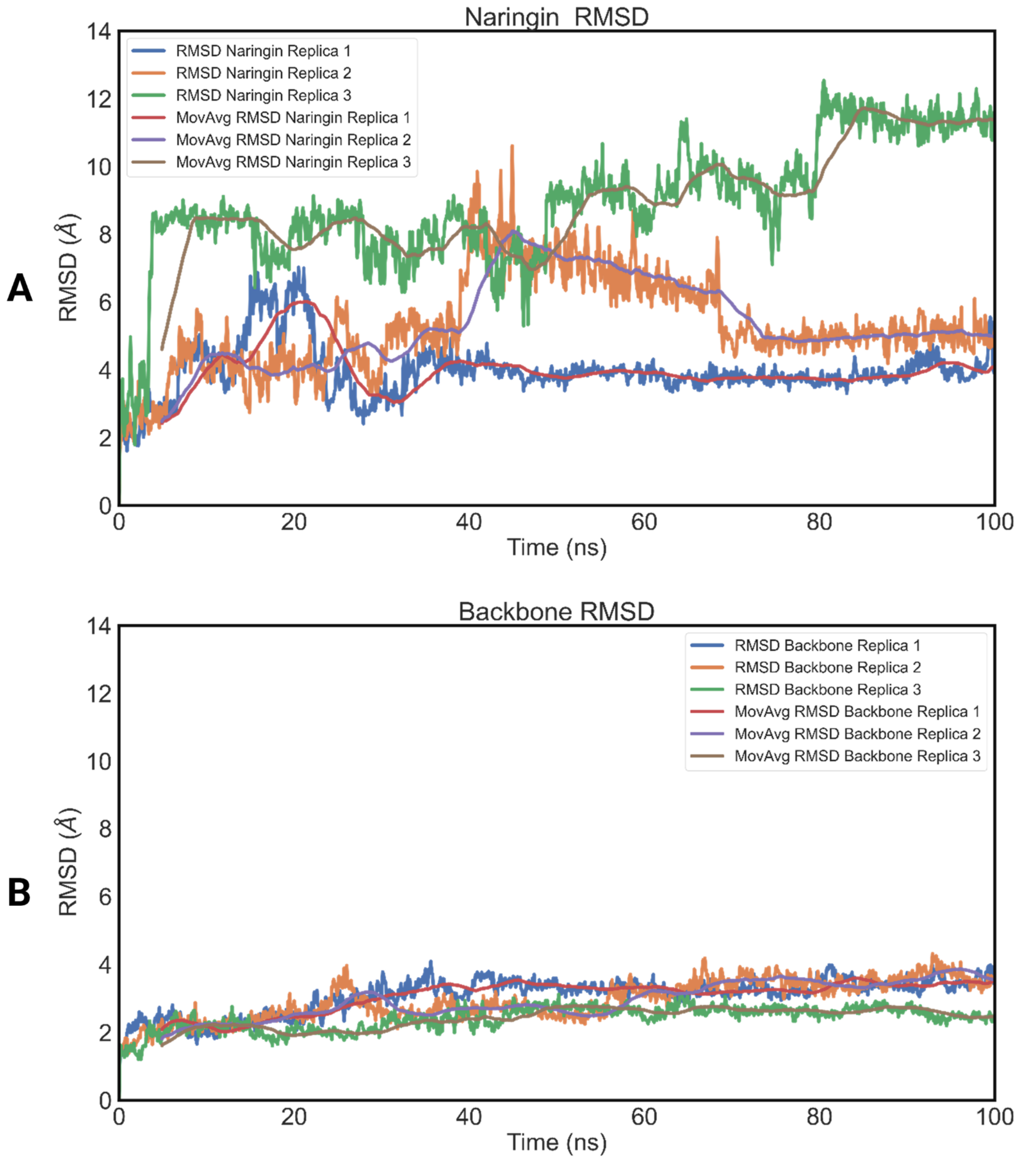

Figure S16. A) RMSD of the three replicas of naringin bound to JNK3 as a function of time, B) RMSD of the three replicas of the protein backbone of JNK3 as a function of time.

Table S13. Average values of RMSD and standard deviation of the protein backbone for each JNK3-ligand complex were calculated over the 100 ns MD simulations.

Replica ID	RMSD quercetin (Å)	RMSD naringin (Å)
1	2.47 ± 0.31	3.07 ± 0.52
2	2.36 ± 0.25	2.94 ± 0.59
3	2.50 ± 0.46	2.41 ± 0.36
Average RMSD	2.45 ± 0.08	2.81 ± 0.35

References

[1] EFSA Panel on Additives and Products or Substances used in Animal Feed (FEEDAP), "Scientific Opinion on the safety and efficacy of naringin when used as a sensory additive for all animal species," *EFSA J.*, vol. 9, no. 11, p. 2416, 2011, doi: 10.2903/j.efsa.2011.2416.

[2] EFSA Panel on Additives and Products or Substances used in Animal Feed (FEEDAP) *et al.*, "Assessment of the feed additive consisting of naringin for all animal species for the renewal of its authorisation (HealthTech Bio Actives, S.L.U. (HTBA))," *EFSA J.*, vol. 20, no. 4, p. e07267, 2022, doi: 10.2903/j.efsa.2022.7267.

[3] EFSA Panel on Additives and Products or Substances used in Animal Feed (FEEDAP) *et al.*, "Safety of vitamin B12 (in the form of cyanocobalamin) produced by Ensifer adhaerensCNCM-I 5541 for all animal species," *EFSA J.*, vol. 18, no. 12, p. e06335, 2020, doi: 10.2903/j.efsa.2020.6335.

[4] EFSA Panel on Dietetic Products, Nutrition and Allergies (NDA), "Scientific Opinion on the substantiation of health claims related to rutin and improvement of endothelium-dependent vasodilation (ID 1649, 1783) and protection of DNA, proteins and lipids from oxidative damage (ID 1784) pursuant to Article 13(1) of Regulation (EC) No 1924/2006," *EFSA J.*, vol. 8, no. 10, p. 1751, 2010, doi: 10.2903/j.efsa.2010.1751.

[5] EFSA Panel on Dietetic Products, Nutrition and Allergies (NDA), "Safety of synthetic trans-resveratrol as a novel food pursuant to Regulation (EC) No 258/97," *EFSA J.*, vol. 14, no. 1, p. 4368, 2016, doi: 10.2903/j.efsa.2016.4368.

[6] EFSA Panel on Dietetic Products, Nutrition and Allergies (NDA), "Scientific Opinion on Dietary Reference Values for folate," *EFSA J.*, vol. 12, no. 11, p. 3893, 2014, doi: 10.2903/j.efsa.2014.3893.

[7] EFSA Panel on Dietetic Products, Nutrition and Allergies (NDA), "Scientific Opinion on the substantiation of a health claim related to a combination of diosmin, troxerutin and hesperidin and maintenance of normal venous tone pursuant to Article 13(5) of Regulation (EC) No 1924/2006," *EFSA J.*, vol. 12, no. 1, p. 3512, 2014, doi: 10.2903/j.efsa.2014.3512.

[8] EFSA Panel on Additives and Products or Substances used in Animal Feed (FEEDAP) *et al.*, "Safety and efficacy of a feed additive consisting of an aqueous extract of Citrus limon (L.) Osbeck (lemon extract) for use in all animal species (Nor-Feed SAS)," *EFSA J.*, vol. 19, no. 11, p. e06893, 2021, doi: 10.2903/j.efsa.2021.6893.

[9] EFSA Panel on Food Additives and Flavourings (FAF) *et al.*, "Re-evaluation of neohesperidine dihydrochalcone (E 959) as a food additive," *EFSA J.*, vol. 20, no. 11, p. e07595, 2022, doi: 10.2903/j.efsa.2022.7595.

[10] EFSA Panel on Food Additives and Nutrient Sources added to Food (ANS) *et al.*, "Scientific opinion on the safety of green tea catechins," *EFSA J.*, vol. 16, no. 4, p. e05239, 2018, doi: 10.2903/j.efsa.2018.5239.

[11] EFSA Panel on Dietetic Products, Nutrition and Allergies (NDA), "Scientific Opinion on the Tolerable Upper Intake Level of eicosapentaenoic acid (EPA), docosahexaenoic acid (DHA) and docosapentaenoic acid (DPA)," *EFSA J.*, vol. 10, no. 7, p. 2815, 2012, doi: 10.2903/j.efsa.2012.2815.

[12] EFSA Panel on Dietetic Products, Nutrition and Allergies (NDA), "Scientific Opinion on the substantiation of a health claim related to alpha linolenic acid and contribution to brain and nerve tissue development pursuant to Article 14 of Regulation (EC) No 1924/2006," *EFSA J.*, vol. 9, no. 4, p. 2130, 2011, doi: 10.2903/j.efsa.2011.2130.

[13] EFSA Panel on Food Additives and Nutrient Sources added to Food (ANS), "Statement on the safety of β-carotene use in heavy smokers," *EFSA J.*, vol. 10, no. 12, p. 2953, 2012, doi: 10.2903/j.efsa.2012.2953.

[14] EFSA Panel on Dietetic Products, Nutrition and Allergies (NDA) *et al.*, "Safety of hydroxytyrosol as a novel food pursuant to Regulation (EC) No 258/97," *EFSA J.*, vol. 15, no. 3, p. e04728, 2017, doi: 10.2903/j.efsa.2017.4728.

[15] M. Bellumori, L. Cecchi, M. Innocenti, M. L. Clodoveo, F. Corbo, and N. Mulinacci, "The EFSA Health Claim on Olive Oil Polyphenols: Acid Hydrolysis Validation and Total Hydroxytyrosol and Tyrosol Determination in Italian Virgin Olive Oils," *Molecules*, vol. 24, no. 11, Art. no. 11, Jan. 2019, doi: 10.3390/molecules24112179.

[16] EFSA Panel on Dietetic Products, Nutrition and Allergies (NDA), "Scientific Opinion on the substantiation of a health claim related to olive (Olea europaea L.) leaf water extract and increase in glucose tolerance pursuant to Article 13(5) of Regulation (EC) No 1924/2006," *EFSA J.*, vol. 12, no. 5, p. 3655, 2014, doi: 10.2903/j.efsa.2014.3655.

[17] EFSA Panel on Dietetic Products, Nutrition and Allergies (NDA), "Scientific Opinion Part I on the substantiation of health claims related to various food(s)/food constituent(s) not supported by pertinent human data (ID 411, 559, 1174, 1184, 1197, 1380, 1409, 1656, 1667, 1670, 1763, 1767, 1806, 1884, 1908, 1997, 2141, 2159, 2243, 2244, 2325, 2331, 2333, 2336, 2652, 2717, 2727, 2752, 2788, 2861, 2870, 2885, 2894, 3077, 3101, 3516, 3595, 3726, 4252, 4288, 4290, 4406, 4509, 4709) pursuant to Article 13(1) of Regulation (EC) No 1924/2006," *EFSA J.*, vol. 9, no. 6, p. 2246, 2011, doi: 10.2903/j.efsa.2011.2246.

[18] T. Dodier, K. L. Anderson, J. Bothwell, J. Hermann, E. A. Lucas, and B. J. Smith, "U.S. Montmorency Tart Cherry Juice Decreases Bone Resorption in Women Aged 65–80 Years," *Nutrients*, vol. 13, no. 2, Art. no. 2, Feb. 2021, doi: 10.3390/nu13020544.

[19] M. J. Ornstrup, T. Harsløf, T. N. Kjær, B. L. Langdahl, and S. B. Pedersen, "Resveratrol Increases Bone Mineral Density and Bone Alkaline Phosphatase in Obese Men: A Randomized Placebo-Controlled Trial," *J. Clin. Endocrinol. Metab.*, vol. 99, no. 12, pp. 4720–4729, Dec. 2014, doi: 10.1210/jc.2014-2799.

[20] A. Alvarez-Sala *et al.*, "A positive impact on the serum lipid profile and cytokines after the consumption of a plant sterol-enriched beverage with a milk fat globule membrane: a clinical study," *Food Funct.*, vol. 9, no. 10, pp. 5209–5219, Oct. 2018, doi: 10.1039/C8FO00353J.

[21] M. J. De Souza *et al.*, "Low Dose Daily Prunes Preserve Hip Bone Mineral Density With No Impact on Body Composition in a 12-Month Randomized Controlled Trial in Postmenopausal Women: The Prune Study," *Curr. Dev. Nutr.*, vol. 6, p. 10, Jun. 2022, doi: 10.1093/cdn/nzac047.010.

[22] C.-L. Shen *et al.*, "Green tea polyphenols supplementation and Tai Chi exercise for postmenopausal osteopenic women: safety and quality of life report," *BMC Complement. Altern. Med.*, vol. 10, no. 1, p. 76, Dec. 2010, doi: 10.1186/1472-6882-10-76.

[23] J. Wattanathorn, W. Somboonporn, S. Sungkamanee, W. Thukummee, and S. Muchimapura, "A Double-Blind Placebo-Controlled Randomized Trial Evaluating the Effect of Polyphenol-Rich Herbal Congee on Bone Turnover Markers of the Perimenopausal and Menopausal Women," *Oxid. Med. Cell. Longev.*, vol. 2018, p. e2091872, Nov. 2018, doi: 10.1155/2018/2091872.

[24] M. N. T. Lambert *et al.*, "Combined bioavailable isoflavones and probiotics improve bone status and estrogen metabolism in postmenopausal osteopenic women: a randomized controlled trial," *Am. J. Clin. Nutr.*, vol. 106, no. 3, pp. 909–920, Mar. 2017, doi: 10.3945/ajcn.117.153353.

[25] L. L. DeBar *et al.*, "YOUTH: A Health Plan–Based Lifestyle Intervention Increases Bone Mineral Density in Adolescent Girls," *Arch. Pediatr. Adolesc. Med.*, vol. 160, no. 12, pp. 1269–1276, Dec. 2006, doi: 10.1001/archpedi.160.12.1269.

[26] J. W. Pawlowski *et al.*, “Impact of equol-producing capacity and soy-isoflavone profiles of supplements on bone calcium retention in postmenopausal women: a randomized crossover trial12,” *Am. J. Clin. Nutr.*, vol. 102, no. 3, pp. 695–703, Sep. 2015, doi: 10.3945/ajcn.114.093906.

[27] “Clinical outcomes of a 2-y soy isoflavone supplementation in menopausal women,” *Am. J. Clin. Nutr.*, vol. 93, no. 2, pp. 356–367, Feb. 2011, doi: 10.3945/ajcn.110.008359.

[28] E. Brink, V. Coxam, S. Robins, K. Wahala, A. Cassidy, and F. Branca, “Long-term consumption of isoflavone-enriched foods does not affect bone mineral density, bone metabolism, or hormonal status in early postmenopausal women: a randomized, double-blind, placebo controlled study2,” *Am. J. Clin. Nutr.*, vol. 87, no. 3, pp. 761–770, Mar. 2008, doi: 10.1093/ajcn/87.3.761.

Disclaimer/Publisher’s Note: The statements, opinions and data contained in all publications are solely those of the individual author(s) and contributor(s) and not of MDPI and/or the editor(s). MDPI and/or the editor(s) disclaim responsibility for any injury to people or property resulting from any ideas, methods, instructions or products referred to in the content.
